# Norwegian Version of the Chelsea Critical Care Physical Assessment Tool (CPAx-NOR): Translation, Face Validity, Cross-Cultural Adaptation and Inter-Rater Reliability

**DOI:** 10.3390/jcm12155033

**Published:** 2023-07-31

**Authors:** Charlotte Marie Schanke, Anne Kristine Brekka, Stein Arne Rimehaug, Mari Klokkerud, Tiina Maarit Andersen

**Affiliations:** 1 Regional Rehabilitation Knowledge Center in South East Norway, 1453 Nesodden, Norway; stein.arne.rimehaug@sunnaas.no (S.A.R.); mariklok@oslomet.no (M.K.); 2 Department of Physiotherapy, Sorlandet Hospital, 4838 Arendal, Norway; anne.kristine.brekka@sshf.no; 3 Department of Thoracic Medicine, Haukeland University Hospital, 5021 Bergen, Norway; tiina.maarit.andersen@helse-bergen.no; 4 Department of Rehabilitation Science and Health Technology, Oslo Metropolitan University, 0130 Oslo, Norway; 5 Faculty of Health and Social Sciences, Bergen University College, 5063 Bergen, Norway

**Keywords:** physiotherapy, physical function, early rehabilitation, measurement tool, critical illness, CPAx, critical care

## Abstract

Background: Assessment of physical and respiratory function in the intensive care unit (ICU) is useful for developing an individualized treatment plan and evaluating patient progress. There is a need for measurement tools that are culturally adapted, reliable and easy to use. The Chelsea Critical Care Physical Assessment Tool (CPAx) is a valid measurement tool with strong psychometric properties for the intensive care population. This study aims to translate, adapt and test face validity and inter-rater reliability of the Norwegian version of CPAx (CPAx-NOR) for use in critically ill adult patients receiving prolonged mechanical ventilation. Method: CPAx-NOR was forward backward translated, culturally adapted and tested by experts and patients for face validity. Thereafter tested by 10 physiotherapists in five hospitals for inter-rater reliability. Results: The experts and pilot testers reached consensus on the translation and face validity. Patients were tested at time point A (n = 57) and at time point B (n = 53). The reliability of CPAx-NOR at “A” was 0.990 (0.983–0.994) and at “B” 0.994 (0.990–0.997). Based on A+B combined and adjusted, the ICC was 0.990 (95% CI 0.996–0.998). Standard error of measurement (SEM) was 0.68 and the minimal detectable change (MDC) was 1.89. The Bland–Altman plot showed low bias and no sign of heteroscedasticity. CPAx-NOR changed with a mean score of 14.9, and showed a moderate floor effect at the start of physiotherapy and low ceiling effects at discharge. Conclusion: CPAx-NOR demonstrated good face validity and excellent inter-rater reliability. It can be used as an assessment tool for physical function in critically ill adults receiving prolonged mechanical ventilation in Norway.

## 1. Introduction

Intensive care unit–acquired weakness (ICU-AW) is common, and if patients survive, it negatively affects quality of life [1] and leads to continuing physical, cognitive and mental impairments [2,3,4,5]. Early rehabilitation starting in the ICU seems to both prevent ICU-AW and improve rehabilitation outcomes [6]. Assessment of several aspects of physical function is essential when developing a treatment plan and evaluating patient progress, as well as to ensure continuity of care from the ICU to the hospital ward [7,8,9]. Physiotherapists’ main responsibility in the multidisciplinary ICU team is to assess and improve the patients respiratory- and general physical function [7,8,9]. Many measurement tools with adequate psychometric properties have been developed for use with ICU patients [10]; however, most of these lack important relevant aspects with regards to respiratory and cough function.

The Chelsea Critical Care Physical Assessment Tool (CPAx) is an observation-based measurement tool developed by Dr. Evelyn Corner. The tool is unique as it incorporates assessment of respiratory function and cough, and both functional and specific muscle testing [10,11,12]. CPAx is valid for the intensive care population and has been translated and tested in different languages, including Danish, Swedish, German and Chinese. It has demonstrated strong psychometric properties and excellent inter-rater reliability in all translations [13,14,15,16]. Considering these aspects CPAx-NOR is minding an important gap in early rehabilitation in critically ill patients in Norway.

To make the measurement tool available and ready for implementation in Norway, it is necessary to agree on a translated and adapted Norwegian version and to test its reliability and ability to detect changes in physical function. It is important to investigate systematic and random errors and establish the minimal detectable change to make the Norwegian version a reliable outcome measure in a Norwegian ICU population.

The aims of this study were to translate, cross-culturally adapt and test face validity of the Chelsea Critical Care Physical Assessment Tool into Norwegian (CPAx-NOR) and to test its inter-rater reliability in critically ill adult patients receiving prolonged mechanical ventilation.

## 2. Materials and Methods

The study had two stages:

Stage I (August 2021–January 2022): Translation, discussions on face validity and cross-cultural adaption of CPAx to Norwegian, and

Stage II (February 2022–September 2022): Evaluation of CPAx-NOR’s inter-rater reliability

The reporting of this study has been structured according to the STROBE recommendations for observational studies [17].

### 2.1. Chelsea Critical Care Physical Assessment—CPAx

CPAx consists of ten different items graded from 0 (unable/dependent) to 5 (independent) on a Guttman scale. The ten items are summarized in an aggregated total score, which indicates the total need for help with a minimum score of 0 (completely dependent) and a maximum score of 50 (independent). The patient is observed and assessed bedside, and the only equipment needed is a handheld dynamometer for measuring grip strength. The use of CPAx is considered feasible in clinical practice, and its visual display makes it easy to understand for both healthcare professionals and patients [11].

#### 2.1.1. Stage I. Translation and Cross-Cultural Adaption

**Figure 1:**
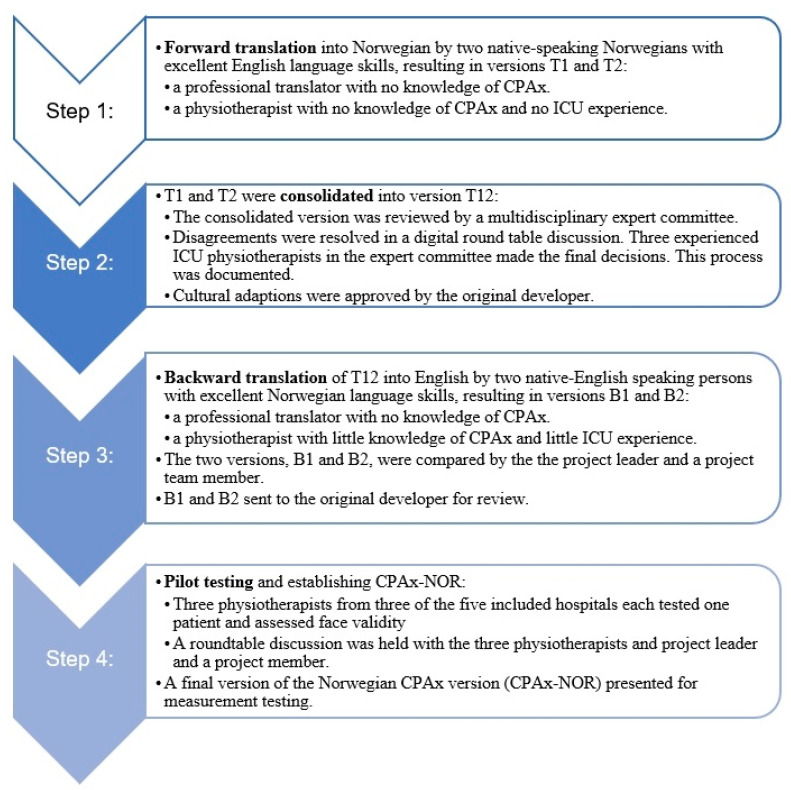
Stage I. The translation process.

Based on international recommendations [18,19,20], a step-by-step forward-backward translation including cross-cultural adaptation with a multidisciplinary expert committee was conducted. The CPAx-NOR was completed in agreement with the original developer, Dr. Evelyn Corner. The process is illustrated in Figure 1 (Step 1 to 3).

As rehabilitation is a multidisciplinary process in the ICU, it was important to ensure that CPAx-NOR was easy to understand both for multidisciplinary teams and for patients. The expert committee members, eight persons, were therefore carefully chosen from hospitals in the South-East health region to involve a broad professional environment. The committee consisted of one senior ICU nurse PhD, one anesthesiologist, one former intensive care patient and five physiotherapists. Three of the physiotherapists had long experience in ICU (>10 years), MSc and specialization in ICU physiotherapy (including two of the authors, CMS and AKB). The other physiotherapists had little ICU experience, whereas one was newly educated. The physiotherapists in the expert committee did not participate in the data collection to test reliability.

The preliminary CPAx-NOR (T12) was then tested in a pilot conducted by another three physiotherapists employed at three of the included hospitals. The physiotherapists: one male and two female with experience ranging from 2–15 years, were not involved in the translation process. They tested one patient each. During a roundtable discussion with the three physiotherapists and CMS and AKB, the final version of CPAx-NOR was agreed upon with one minor change (Figure 1, Step 4).

#### 2.1.2. Face Validity

Assessment of face validity as described by COSMIN [21] was conducted by considering the relevance, purpose and whether the items reflected the construct to be measured in discussions between physiotherapists, anesthesiologist, nurse and former ICU patient (Figure 1, step 2) and physiotherapists, patients, and the project leader and a project member (Figure 1, Step 4).

### 2.2. Stage II. Evaluation of CPAx-NOR Inter-Rater Reliability

#### Design and Setting

A multicentre study with a prospective cross-sectional design was conducted in five hospitals in Norway’s South-East health region including both large university hospitals and smaller local hospitals. All the units were general ICUs and included 33 ICU beds at the time of the inclusion period. The study was presented to the Regional Ethics Committee, which concluded that it did not require approval. The Data Inspectorate at all the local hospitals and SIKT (Norwegian Agency for Shared Services in Education and Research, formerly the Norwegian Centre for Research Data), approved the study, project number 777606. The study was conducted according to the Helsinki Declaration, and all participants (physiotherapists and patients) gave informed, written consent before inclusion in the study.

### 2.3. Participants and Patients

The aim of this part of the study was to investigate inter-rater reliability and therefore the participants of interest were the physiotherapists using CPAx-NOR, and how they used CPAx-NOR to rate patients in clinical practice in the ICU. Ten physiotherapists (one man and nine women), age 28 to 64 years, participated as testers for CPAx-NOR. Six of them were specialists in ICU physiotherapy. They all had more than four years clinical experience from a hospital, and their clinical experience at the ICU ranged from one to more than 30 years. None of the physiotherapists had used CPAx routinely prior to the study.

The median length of stay in Norwegian ICUs is 2.1 days and median time on mechanical ventilation is 1.5 days [22]. Therefor the physiotherapists included and tested patients according to the following criteria: The patients had to be referred to physiotherapy at the ICU. Adults (age > 18 years) who were mechanically ventilated ≥48 h during their stay in the ICU were considered at risk of ICU-AW and included. No exclusion criteria were set. 

### 2.4. Data Collection, Procedures and Measurement

The only demographic data collected were age, sex, diagnosis and CPAx-NOR score.

All the participating physiotherapists completed the same education to use CPAx-NOR in clinical practice. The original English eLearning platform used in previous studies was not available, therefore a six-hour digital course in Norwegian was developed and discussed in agreement with the original CPAx developer, Evelyn Corner. It consisted of three parts, (1) a theory part on the use of standardized measurement tools in general, (2) a comprehensive review of the CPAx, (3) an instructional video of a physiotherapy treatment session with a simulated patient to be scored and discussed in a plenary setting. At each hospital, two physiotherapists completed all the assessments at the same time in pairs with separate roles; one conducted the assessment while the other observed. They both scored separately and blinded. The testers decided the roles without further instructions from the project leader. To facilitate patient inclusion and to help with practical problem-solving during testing of CPAx-NOR, monthly digital meetings were arranged for all the participating physiotherapists in combination with an open invitation to correspond via e-mail, and one in-person meeting at each participating hospital.

### 2.5. Inter-Rater Reliability

Inter-rater reliability was assessed at start of physiotherapy and at discharge from ICU, according to COSMIN criteria [23], and a sample size of at least 50 patient scoring was considered sufficient for inter-rater reliability testing [24]. It is important to establish reliability before measurement instruments can be used in clinical practice as reliability refers to the extent the measurement can be replicated [25]. We hypothesized that CPAx-NOR would show good inter-rater reliability of aggregated scores and individual items with an ICC > 0.80 and weighted kappa values >0.81.

### 2.6. Change in Scores during Patient Trajectory

Change in scores, understood as CPAx-NOR’s ability to detect change in a patient trajectory in the ICU unit over time, was described by effect size (ES) and standard response mean (SRM) [26,27].

### 2.7. Statistical Analysis

Descriptive and non-parametric statistics were used to describe the demographic characteristics and distribution of scores, expressed as the mean, standard deviation (SD), median, interquartile range (IQR), frequency and percentage of the data. To analyze the inter-rater reliability, we used the intraclass correlation coefficient (ICC) with 95% confidence intervals for aggregated scores. Because this was a multicenter study, and not every patient was rated by each rater, we used a one-way random effects model (single measurement) as described in Koo and Li [25] and Shrout and Fleiss [28] at each of the two time points (A and B) and for both visits. Since CPAx is an ordinal scale and absolute disagreements between raters are investigated, it is important to give different weights to the size of disagreements using quadratic weighted Cohen’s kappa for individual items [24,29]. The standard error of measurement (SEM = SD × (sqr 1 − ICC) and minimal detectable change (MDC) (=SEM × 1.96 × √2) with limits of agreement (LOA) were calculated as parameters of measurement error for aggregated scores at both measurement points. SEM was considered acceptable if equal to the original, ≤3 [30]. Limits of agreement (LOA) were defined as d±1.96×SDdiff where d = mean difference between raters and SDdiff = the standard deviation of the differences. A Bland–Altman plot was used to visualize the scores and to look for outliers, systematic bias and heteroscedasticity [31] on each time point and total scores were corrected for repeated measures using the ‘true value varies’ method [32]. The measurement error for individual items was calculated and displayed as percentage agreement. To calculate effect size (ES) we used the mean of aggregated discharge score minus the mean of aggregated initiation of physiotherapy score divided by the SD of mean initiation score. To calculate the standardized response mean (SRM) we used the mean of aggregated discharge score minus the mean of aggregated initiation of physiotherapy score divided by the SD of mean difference [26]. Floor and ceiling effects are reported as the percentage of patients scoring zero or fifty at the two measurement points and are considered acceptable if <15% [24]. Data were stored and processed in IBM SPSS version 28.0.1 for Windows or Microsoft Excel.

## 3. Results

### 3.1. Translation, Cross-Cultural Adaption and Face Validity

The CPAx-NOR was translated in a step-by-step protocol shown in Figure 1. The expert committee discussed several minor cultural and linguistic differences, and the original developer approved all the adjustments. There was a need to clarify the item ‘cough’. Deep suction was defined as suction below the cannula. Further, the term ‘Yankauer suction’ is not used in Norway and was described instead as ‘suction in the mouth and upper throat’. Another important clarification was the rating of patients’ physical function based on actual performance with the need for ‘minimal, moderate or maximal’ assistance. After the adjustments, all the participants in the expert committee agreed on the preliminary CPAx-NOR version to be tested in clinical practice. The pilot testing demonstrated that the preliminary CPAx-NOR was feasible for use and valid with one minor adjustment. The Norwegian CPAx was established. Both the expert committee and the physiotherapists and patients in the pilot testing, agreed that the items in CPAx-NOR was relevant and reflected the constructs to be measured in an adult patient population in ICU, thereby demonstrating good face validity. The final version of CPAx-NOR is located in Supplementary Materials.

### 3.2. Patient Population at Start of Physiotherapy (A) and at Discharge from the ICU (B)

**Figure 2:**
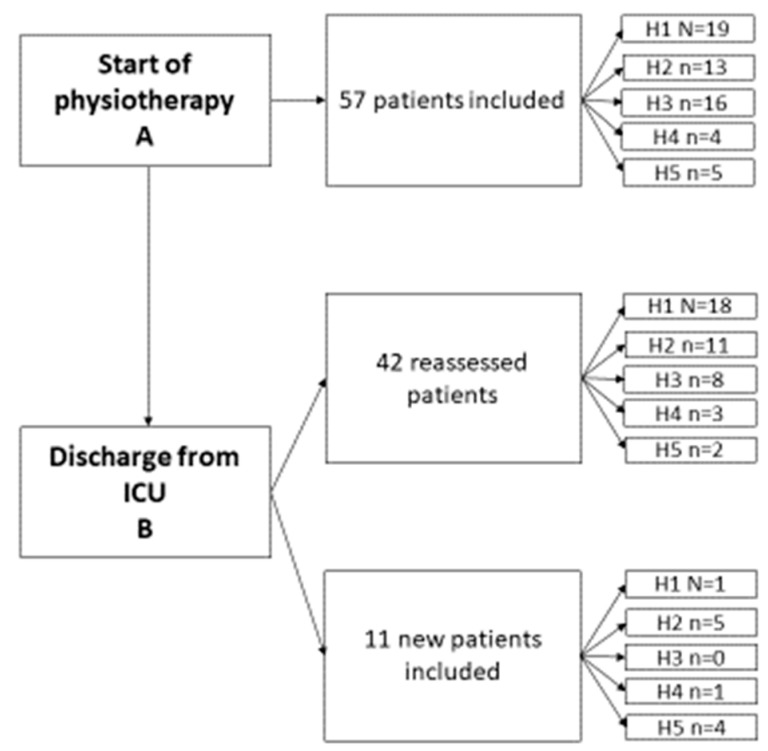
Flow chart showing patient inclusion in the 5 different hospitals (H1-H5) during the study.

After the CPAx-NOR was established and the education completed, the five hospitals started including patients from their ICUs, see Figure 2. From February 1 until the end of September 2022, 57 patients (23 women), mean age 64 years, were included at time point A—start of physiotherapy. At point B—discharge from ICU—53 patients (20 women), mean age 64 years, were included. See Table 1 for further details. The patients were divided into five diagnostic groups representative of the intensive care population in Norway [22]. No informed consent was withdrawn in this study.

### 3.3. Inter-Rater Reliability and Limits of Agreement

The ICC was 0.990 (0.983–0.994) at time point A and 0.994 (0.990–0.997) at time point B. The ICC for both time points combined (A+B) was 0.998, SEM 0.68 and the MDC was 1.89. See Table 2.

**Figure 3:**
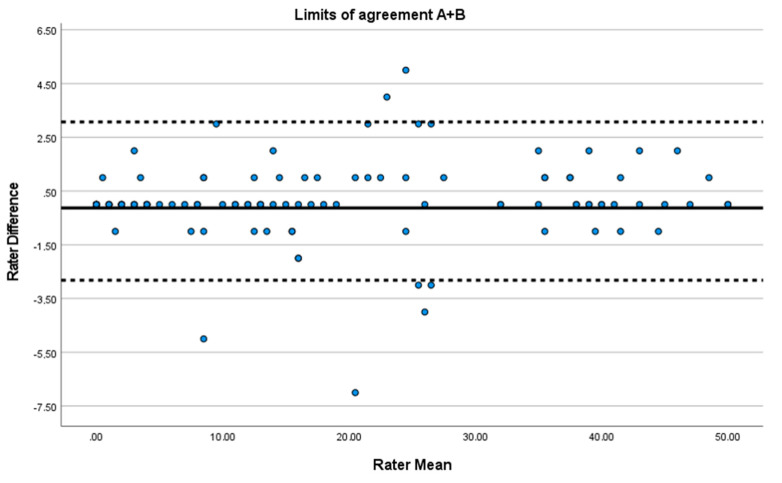
Modified Bland–Altman plot of data adjusted for repeated measurements. Limits of agreement were defined as d±1.96×SDdiff where d  = mean difference between raters and SDdiff = the standard deviation of the differences. The mean CPAx-NOR score is indicated with a solid line and the upper and lower limits are indicated with dotted lines.

The Bland–Altman plot for the total scores combined shows the mean difference between raters was −0.13 (SD 1.50) and 95% limits of agreement were from −2.82 to 3.07. The limits of agreement are shown in Figure 3.

**Figure 4:**
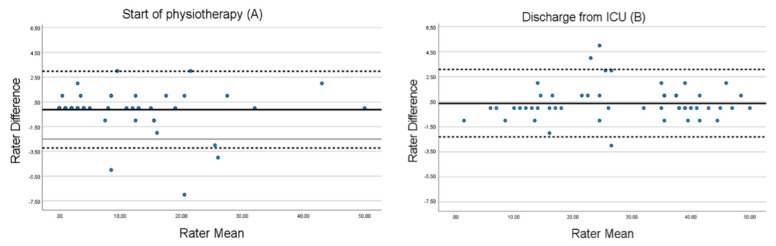
Bland–Altman plot of data from 57 assessments of critically ill patients at time point A and 53 assessments at time point B. The mean is indicated with a solid line and the upper and lower limits are indicated with dotted lines.

At time point A, the mean difference between raters was −0.12 (SD 1.58) and 95% limits of agreement was −3.22 to 2.98. At time point B, the mean difference between raters was 0.40 (SD 1.38) and 95% limits of agreement was −2.31 to 3.09. The limits of agreement are shown in Figure 4.

The ten individual items showed weighted kappa values between 0.957 and 0.996 at time point A and between 0.925 and 0.980 at time point B. The percentage agreement for individual items, as a parameter for measurement error, ranged from 77.2% to 98.2% at time point A and 73.6% to 98.1% at time point B. Results are presented in Table 3.

### 3.4. Change in Scores of CPAx-NOR

The mean difference in CPAx-NOR score between time points A and B (n = 42) was 14.9 (95% CI 11.0; 18.7). ES was 1.3 and SRM was calculated as 1.2.

### 3.5. Floor and Ceiling Effects

Nine patients scored 0 (16%) and one patient scored 50 (1.8%) at the start of physiotherapy (median 5, IQR 2–15.5; range 0–50). At ICU discharge, none of the patients scored 0. One patient scored 50 points (2%) (median 28, IQR 15.5–39.5; range 1–50). These results indicate that problems related to floor effects may be moderate while problems related to ceiling effects are minimal.

## 4. Discussion

The objective of this study was to translate, adapt, test face validity and the inter-rater reliability of CPAx-NOR in critically ill adult patients undergoing prolonged mechanical ventilation in the ICU. CPAx-NOR was successfully translated in a forward-backward translation, cross-culturally adapted and pilot tested in clinical practice. Face validity was assessed through expert group and patient discussions, and demonstrated good results. CPAx-NOR demonstrated excellent inter-rater reliability for both aggregated score and all ten individual items across the ICU stay. The measurement error was small with a minimal detectable change of two points. CPAx-NOR exhibited a moderate floor effect at start of physiotherapy and low floor and ceiling effects at ICU discharge, as expected. These results indicate that CPAx-NOR is a valid and reliable measurement tool for physical function during the continuum of an ICU stay for adult patients on mechanical ventilation for more than 48 h. Whether the results are generalizable to other ICU patients with shorter time on mechanical ventilation has not been investigated.

### 4.1. Reliability

The MDC is of great importance when evaluating change in physical function. Previous studies investigating CPAx [13,14,30], have reported the SEM and MDC at only one time point. However, in the German version MDC has been reported on several time points [15]. The present study measured SEM and MDC at the two time points and showed that if two different raters assess the patient early in the ICU stay (at the start of physiotherapy), a change of >3 points indicate a detectable/true change in physical function above the measurement error. Later in the patient trajectory, at measurement point B, when a higher level of functioning is present, a change of >2 points indicated a detectable change in physical function. This is an important consideration when evaluating the patients’ rehabilitation process throughout the patient trajectory when not only one, but several physiotherapists perform assessments. These results are similar to the Danish version [13]. When the scores of the time points were combined (A+B), we also found that a change of 2 points indicated a detectable change in physical function, similar to the German version [15]. Different MDC across the measurement tool’s scale has also been established in other measurement tools such as the Bergs Balance Scale [33]. The differences in the MDC reported in CPAx studies [13,14,15,30] may be related to the defined time point for the assessment and the selected method of reporting this psychometric. This needs to be standardized when designing future international studies.

Different MDC’s corresponds to the finding that the agreement was somewhat lower for the items ‘cough’ (77.2% and 79.2%), ‘moving within the bed’ (86.0% and 73.6%) and ‘dynamic sitting’ (87.7% and 79.2%). Despite the clarification of the item ‘cough’ during the translation, disagreements among the raters were still present at both time points. Suctioning is a part of the item ‘cough’, and in Norway, this task is assigned to nurses, which might have complicated the evaluation. The minimal, moderate and maximal assistance ratings were the subject of several discussions between clinicians during the testing period and may be the reason for the lower agreement on these items, similar to findings from previous publications using CPAx [12,13,14]. This underpins the need to develop local standardized recommendations prior to the implementation of CPAx-NOR in clinical practice.

Our SEM and MDC at time point A are similar to the original version [27], and the agreement between tester and observer was high in general, both on each time point and in total, as shown in the Bland–Altman plot (Figure 2 and Figure 3). These Bland-Altman Plots showed no bias due to the role of the rater during assessments (tester or observer) and no heteroscedasticity. These results correspond to those of other studies that have used this method to illustrate agreement [12,13,14,15].

CPAx-NOR is a clinically useful tool for assessing low-functioning patients receiving prolonged mechanical ventilation in the study population representative of the Norwegian ICU population expressed as the SEM, MDC and LOA throughout the ICU trajectory. The results of previous studies assessing inter-rater reliability, together with the current study, support the claim that CPAx is in general a reliable tool to assess function in the ICU population.

### 4.2. Change in Scores

Our results showed a mean change of 15 points in scores between the start of physiotherapy and discharge from the ICU. This indicates that the patients’ physical function improved during the trajectory, a finding supported by the large ES and SRM. These findings are similar to those in studies of responsiveness of the original and the Danish version [13,30]. Generally, patients had a first visit from a physiotherapist within 72 h, but as as we did not collecta data on this, we can not report median time. Corner [30] established a Minimal Clinical Important Difference (MCID) to be six points, but as this was in a burn population, we cannot directly compare the populations although the change in scores exceeded six points. A specific investigation of both the MCID and the responsiveness of CPAx-NOR including data on length of stay in the ICU needs to be conducted to come to any conclusion on this matter.

### 4.3. Floor and Ceiling Effects

As with the original version of CPAx [30], the floor effect (patients with a total score of 0) was moderate at the start of physiotherapy (16%); all patients with aggregated score 0 died during the study period and the authors suggested the total score of 0 can predict death [30]. In this study, 55.5% of the patients with total score 0, survived the ICU stay and were discharged to a regular ward. We do not have discharge scores for the remaining 44.5% and are not able to make conclusions regarding survival or death for these patients. Thus, the present study does not indicate that a total score of 0 at the start of physiotherapy in Norway predicts death. However, the patients with a total CPAx-NOR score of 0 at the time point A did score less than 12 points at time point B. This aggregated score is below the mean total score in the present study, indicating poorer physical function requiring a higher level of care. This may be useful in predicting what level of care and what degree of rehabilitation intensive care patients need after discharge from hospitals in Norway but needs to be investigated further. Both patients and the healthcare system have a great interest in starting planning early to ensure a seamless rehabilitation process, and CPAx has already demonstrated these qualities as a predictive measurement tool in the original and German versions [34,35].

At time point B, no patients scored 0 points, and only 2% scored 50 points, meaning no floor effect and a highly acceptable ceiling effect. This corresponds with the results reported in the original [30] and three other translations [13,14,15]. These results indicate that CPAx-NOR is applicable for clinical use in Norwegian intensive care units from early in the rehabilitation process through the patient trajectory, including discharge from the ICU to a regular ward.

### 4.4. Perspective, Further Research and Clinical Implications

CPAx-NOR is an important tool in clinical practice to help establish rehabilitation goals for ICU patients, as they fight their way back toward regaining independent respiratory and physical function [11]. Standardized measuring tools are crucial to document the effect of physiotherapy and early rehabilitation, both clinically and in research. Barriers and facilitators to implementing measurement tools should be further investigated, in order to successfully implement the CPAx-NOR and maintain sustainability in clinical practice. Future studies should include also other aspects of validity.

Both the similarities and differences between the original and translated CPAx versions, highlights the importance of a solid translation- and cross-cultural adaptation process that is needed for further standardization when implementing the CPAx versions into clinical practice. These findings are of importance in future research when designing international multicenter studies, with aims to investigate the effect of early rehabilitation at the ICU. Of clinical importance, is how to apply the MDC results of this study. The authors recommend applying the combined result of the two time points A+B (MDC = 2 points) in clinical practice, similar to the Danish and German versions [13,15].

### 4.5. Strengths and Limitations

The strengths of the present study include the comprehensive translation process with two professional translators and two clinicians involved in both the forward and backwards translations, along with a multidisciplinary expert committee including a former intensive care patient, before further evaluation of inter-rater reliability. Due to the multicentre design of this study, with five hospitals considered representative of Norwegian ICUs in terms of size, organization and patient population, the results can be considered generalizable to other ICUs in Norway. Further, the use of raters with a range of experience as physiotherapists in acute hospital settings supports our confidence that the use of CPAx-NOR is feasible for a wide variety of physiotherapists working in ICUs.

As in the other European translation studies, all the raters completed a digital course to the testing period, but unlike in those studies, the original English eLearning platform was not available. All raters received training before assessing patients, and generalizability for physiotherapists without any training is limited. Another possible limitation of the design was that the assessments were completed in pairs with raters alternating between the roles of leader and observer without structure. This made it difficult to identify systematic between-raters error.

Moreover, we did not have data on median time on time point A or on length of stay (ICU LOS) in the patients that were tested. Therefore we were not able to link CPAx-NOR to a specific time point in the patient trajectory and to make any conclusions regarding responsiveness.

## 5. Conclusions

CPAx has been successfully translated and cross-culturally adapted into Norwegian, resulting in CPAx-NOR. The adapted tool has been found to show good face validity in clinical practice and has demonstrated excellent inter-rater reliability. CPAx-NOR can be considered an important measurement instrument for physiotherapists working in the ICU for assessing respiratory and physical function and planning and setting goals for early rehabilitation in the multidisciplinary team in intensive care units in Norway. Future studies should focus on an extended validation, establishing MCID and studying responsiveness in order to insure CPAx-NOR as a clinically important and knowledge based robust tool for physiotherapists working in the intensive care unit with critically ill patients.

## Figures and Tables

**Table 1:** Characteristics of the included patients at time point A—start of physiotherapy, and at time point B—at discharge from the ICU.

Characteristics of the Patient Population Scored with CPAx-NOR	Start of Physiotherapy n = 57 A	Discharge from ICU n = 53 B
Sex, n (%)	Men 34 (n = 60%) Women 23 (n = 40%)	Men 33 (n = 62%) Women 20 (n = 38%)
Age, yrs Mean (range)	64 (24–84)	64 (24–84)
Type of diagnosis, % (n = men/women):		
Cardiovascular	21.1 (8/4)	20.8 (8/3)
Respiratory	28.1 (11/5)	26.4 (10/4)
Infection	21.1 (7/5)	18.9 (5/5)
Postoperative complications	15.8 (3/6)	17.0 (4/5)
Other *	14.0 (5/3)	17.0 (6/3)

* includes neurological, multitrauma, intox etc.

**Table 2:** Inter-rater reliability results of aggregated scores of CPAx-NOR at start of physiotherapy (A), discharge from ICU (B) and A+B.

CPAx-NOR Score	Lead Rater Mean (SD) Min-Max	Observer Rater Mean (SD) Min-Max	ICC (95%CI)	SEM	MDC
Time point A n = 57	9.60 (10.84) 0–50	9.72 (11.00) 0–50	0.990 (0.983–0.994)	0.77	2.12
Time point B n = 53	28.45 (13.24) 1–50	28.06 (13.15) 2–50	0.994 (0.990–0.997)	0.72	2.0
Time point A + B n = 110			0.998 (0.996–0.998)	0.68	1.89

SD: standard deviation; ICC: intraclass correlation coefficient; 95%CI: confidence interval; SEM: standard error of measurement (SEM = SD × (sqr 1 − ICC)); MDC; minimal detectable change (=SEM × 1.96 × √2).

**Table 3:** Inter-rater reliability of individual items of CPAx-NOR in ICU patients at time point A and time point B.

CPAx-NOR Items	Start of Physiotherapy (A) n = 57				Discharge from the ICU (B) n = 53			
	Lead Rater Median (IQR 25–75%)	Observer Rater Median (IQR 25–75%)	Weighted Kappa Values	Absolute Agreement (%)	Lead Rater Median (IQR 25–75%)	Observer Rater Median (IQR 25–75%)	Weighted Kappa Values	Absolute Agreement (%)
Respiratory function	2 (1–4)	2 (1–4)	0.987	93.0	5 (4–5)	5 (4–5)	0.980	98.1
Cough	2 (1–4)	2 (1 4)	0.940	77.2	5 (4–5)	5 (4–5)	0.931	79.2
Moving within the bed	0 (0 1)	0 (0 1)	0.905	86.0	3 (2–4)	3 (1–4)	0.925	73.6
Supine to sitting on the edge of the bed	0 (0–1)	0 (0–1)	0.972	93.0	2 (1–4)	2 (1 4)	0.965	83.0
Dynamic sitting	0 (0–2)	0 (0–2)	0.957	87.7	4 (3–5)	4 (3–5)	0.961	79.2
Standing balance	0 (0–0)	0 (0–0)	0.959	94.7	3 (0–4)	3 (0 4)	0.990	94.3
Sit to stand	0 (0–0)	0 (0–0)	0.967	93.0	2 (0–3)	2 (0–3)	0.980	88.7
Transferring from bed to chair	0 (0–0)	0 (0–0)	0.975	98.2	2 (0–4)	2 (0–4)	0.992	96.2
Stepping	0 (0–0)	0 (0–0)	0.969	96.5	2 (0–4)	2 (0–4)	0.970	88.7
Grip strength	0 (0–2)	0 (0–2)	0.996	98.2	2 (1–3)	2 (1–3)	0.992	96.2

IQR: interquartile range.

## Data Availability

The data presented in this study are available on request from the corresponding author. The data are not publicly available due to privacy and ethical restrictions in the statement from SIKT (Norwegian Agency for Shared Services in Education and Research.

## Supplementary Materials

The following supporting information can be downloaded at: https://www.mdpi.com/article/10.3390/jcm12155033/s1, Norwegian Version of Chelsea Critical Physical Assessment Tool—CPAx NOR. Reference [36] is cited in the supplementary materials.
