# Supplementary material for: Norwegian Version of the Chelsea Critical Care Physical Assessment Tool (CPAx-NOR): Translation, Face Validity, Cross-Cultural Adaptation and Inter-Rater Reliability
Source: J Clin Med. 2023 Jul 31;12(15):5033. doi: 10.3390/jcm12155033 (PMC10419396; doi:10.3390/jcm12155033)
Supplement: Supplementary file 1 [file jcm-12-05033-s001.zip › CPAx-NOR 30.07.23.pdf]

The Chelsea Critical Care Physical Assessment tool, Norwegian version (CPAx-NOR)

FYSISK DELFUNKSJON	NIVÅ 0	NIVÅ 1	NIVÅ 2	NIVÅ 3	NIVÅ 4	NIVÅ 5
**Respiratorisk funksjon.**	Fullstendig avhengig av respirator. Kontrollert modus. Kan være helt sedert/muskelrelaksert.	Avhengig av respirator. Kontrollert modus med noen spontane pust.	Puster selv med kontinuerlig invasiv eller non-invasiv ventilasjonsstøtte.	Puster selv med intermit-terende invasiv eller non-invasiv ventilasjonsstøtte ELLER «High-flow» oksygen-tilskudd (>15 liter).	Får oksygentilskudd (<15 liter)	Selvpustende uten oksygentilskudd.
**Hoste.**	Ingen hoste, kan være helt sedert/muskelrelaksert.	Hoste stimuleres kun av dyp suging.	Svak ineffektiv hoste, uten evne til sekret-evakuering, f.eks. behov for dyp suging.	Svak, delvis effektiv hoste, med sporadisk evne til sekretevakuering, f.eks. behov for sug i munn/svelg.	Effektiv hoste, sekret-evakuering ved hjelp av sekretmobiliserende teknikker.	Effektiv hoste, fjerner sekret selvstendig.
**Forflytning i seng f.eks. snu seg.**	Kan ikke, kan være helt sedert/muskelrelaksert.	Initierer bevegelsen. Må ha hjelp av ≥2 personer (maksimal).	Initierer bevegelsen. Må ha hjelp av ≥1 person (moderat).	Initierer bevegelsen. Må ha hjelp av 1 person (minimal).	Selvstendig på ≥3 sekunder.	Selvstendig på <3 sekunder.
**Liggende til sittende på sengekant.**	Kan ikke/ustabil.	Initierer bevegelsen. Må ha hjelp av ≥2 personer (maksimal).	Initierer bevegelsen. Må ha hjelp av ≥1 person (moderat).	Initierer bevegelsen. Må ha hjelp av 1 person (minimal).	Selvstendig på ≥3 sekunder.	Selvstendig på <3 sekunder.
**Sittebalanse (dvs. sitterpå sengekanten/ sitter uten støtte).**	Kan ikke/ustabil.	Må ha hjelp av ≥2 personer (maksimal).	Må ha hjelp av ≥1 person (moderat).	Må ha hjelp av 1 person (minimal).	Selvstendig med noe dynamisk sittebalanse, dvs. kan endre over- kroppens stilling innenfor understøttelsesflaten.	Selvstendig med full dynamisk sittebalanse, dvs. kan strekke seg ut over understøttelsesflaten.
**Stående balanse.**	Kan ikke/ustabil/sengeleie.	Ståseng eller lignende.	Ståheis eller lignende.	Avhengig av prekestol, rullator, krykker eller lignende.	Selvstendig uten hjelpemidler.	Selvstendig uten hjelpe-midler og full dynamisk stående balanse, dvs. kan strekke seg ut over under-støttelsesflaten.
**Sittende til stående (Utgangsstilling: ≤90 grader hoftefleksjon).**	Kan ikke/ustabil.	Sittende til stående med maksimal hjelp, f.eks. ståheis eller lignende.	Sittende til stående med moderat hjelp, f.eks. 1-2 personer.	Sittende til stående med minimal hjelp, f.eks. 1 person.	Selvstendig fra sittende til stående ved å skyve fra mot armlenene på stolen.	Selvstendig fra sittende til stående uten armbruk.
**Forflytning fra seng til stol.**	Kan ikke/ustabil.	Heis.	Ståheis eller lignende.	Lav forflytning (ingen skritt) med forflytningshjelpe-middel eller fysisk hjelp.	Stående forflytning med skritt med forflytnings-hjelpemiddel ELLER fysisk hjelp.	Selvstendig forflytning uten utstyr.
**Gange.**	Kan ikke/ustabil.	Bruker ståheis eller lignende.	Bruker forflytnings-hjelpemidler OG hjelp av <1 person (moderat).	Bruker forflytnings-hjelpemidler OG hjelp av 1 person (minimal).	Bruker forflytnings-hjelpemiddel ELLER hjelp av 1 person (minimal).	Selvstendig uten hjelp.
**Gripestyrke (predikert gjennomsnitt for alder og kjønn for den sterkeste hånden).**	Kan ikke testes.	<20%	<40%	<60%	<80%	≥80%

REGIONAL KOMPETANSETJENESTE FOR REHABILITERING
Sunnaas sykehus

C. Schanke 01/11/2022 © Copyright of Chelsea and Westminster NHS Foundation Trust (01/03/2010)

The Chelsea Critical Care Physical Assessment tool, Norwegian version (CPAx-NOR)

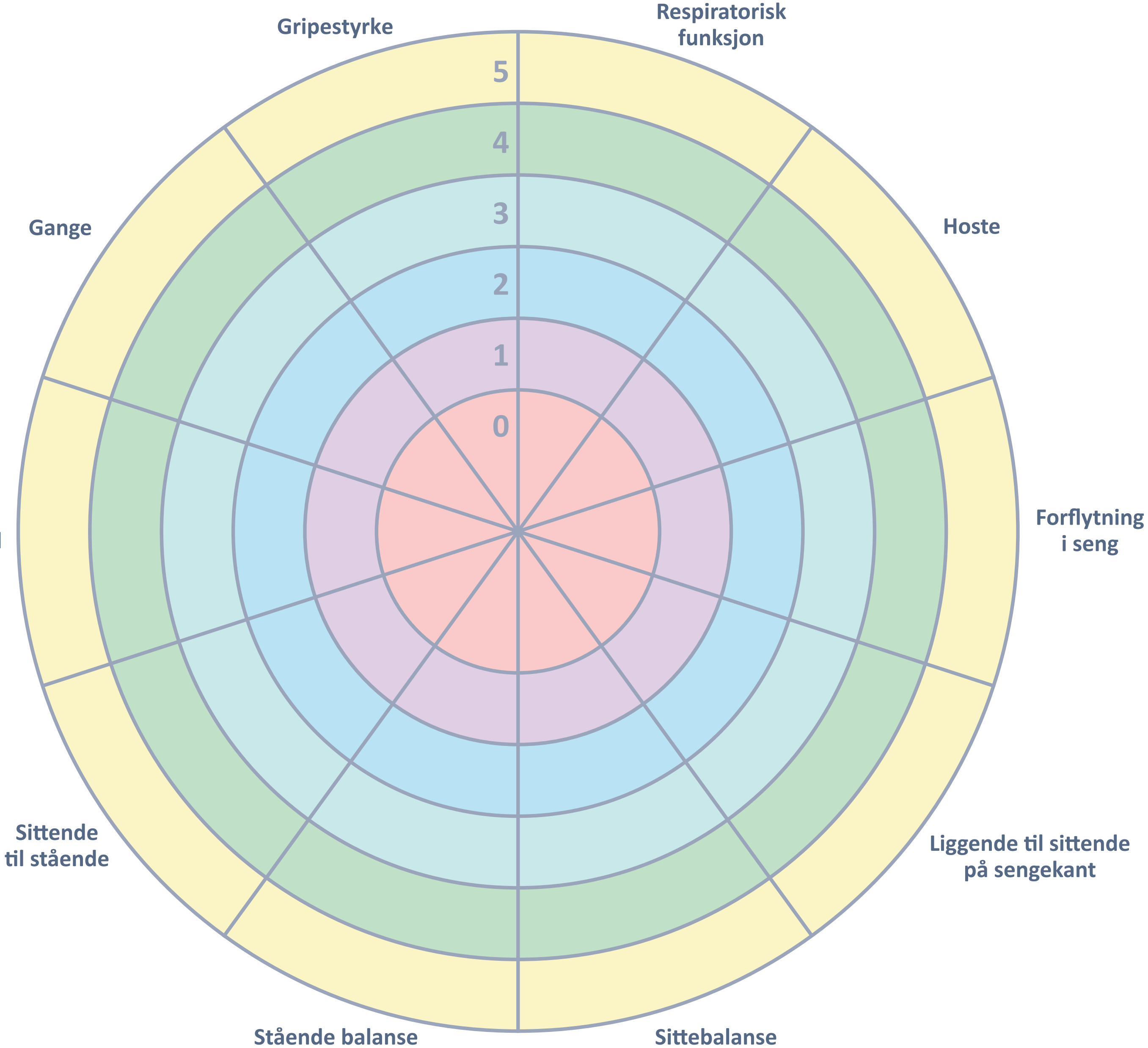

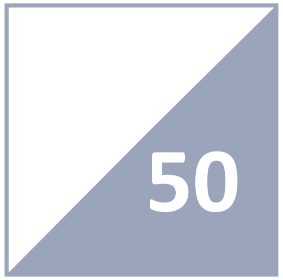

REGIONAL KOMPETANSETJENESTE FOR REHABILITERING
Sunnaas sykehus

C. Schanke 01/11/2022 © Copyright of Chelsea and Westminster NHS Foundation Trust (01/03/2010)

The Chelsea Critical Care Physical Assessment tool, Norwegian version (CPAx-NOR)

ALDER (år)	MENN							KVINNER						
	Hånd	Gjennom-snitt	<20%	<40%	<60%	<80%	≥80%	Hånd	Gjennom-snitt	20%	<40%	<60%	<80%	≥80%
15 til 19	H	46.91	9.38	18.76	28.15	37.53	37.53	H	28.82	5.76	11.53	17.29	23.06	23.06
	V	42.13	8.43	16.85	25.28	33.70	33.70	V	24.98	5.00	9.99	14.99	19.98	19.98
20 til 24	H	48.15	9.63	19.26	28.89	38.52	38.52	H	28.33	5.67	11.33	17.00	22.66	22.66
	V	43.08	8.62	17.23	25.85	34.46	34.46	V	25.78	5.16	10.31	15.47	20.62	20.62
25 til 29	H	53.76	10.75	21.50	32.26	43.01	43.01	H	33.82	6.76	13.53	20.29	27.06	27.06
	V	48.60	9.72	19.44	29.16	38.88	38.88	V	30.31	6.06	12.12	18.19	24.25	24.25
30 til 34	H	52.63	10.53	21.05	31.58	42.10	42.10	H	33.97	6.79	13.59	20.38	27.18	27.18
	V	48.98	9.80	19.59	29.39	39.18	39.18	V	31.64	6.33	12.66	18.98	25.31	25.31
35 til 39	H	53.16	10.63	21.26	31.90	42.53	42.53	H	32.46	6.49	12.98	19.48	25.97	25.97
	V	51.75	10.35	20.70	31.05	41.40	41.40	V	29.77	5.95	11.91	17.86	23.82	23.82
40 til 44	H	55.49	11.10	22.20	33.29	44.39	44.39	H	30.34	6.07	12.14	18.20	24.27	24.27
	V	50.40	10.08	20.16	30.24	40.32	40.32	V	26.23	5.25	10.49	15.74	20.98	20.98
45 til 49	H	49.93	9.99	19.97	29.96	39.94	39.94	H	35.30	7.06	14.12	21.18	28.24	28.24
	V	48.94	9.79	19.58	29.36	39.15	39.15	V	32.06	6.41	12.82	19.24	25.65	25.65
50 til 54	H	48.40	8.68	19.36	29.04	38.72	38.72	H	28.37	5.67	11.35	17.02	22.70	22.70
	V	41.46	8.29	16.58	24.88	33.17	33.17	V	26.28	5.26	10.51	15.77	21.02	21.02
55 til 59	H	45.71	9.14	18.28	27.43	36.57	36.57	H	29.76	5.95	11.90	17.86	23.81	23.81
	V	42.16	8.43	16.86	25.30	33.73	33.73	V	27.81	5.56	11.12	16.69	22.25	22.25
60 til 64	H	40.59	8.12	16.24	24.35	32.47	32.47	H	26.35	5.27	10.54	15.81	21.08	21.08
	V	37.25	7.45	14.90	22.35	29.80	29.80	V	23.47	4.69	9.39	14.08	18.78	18.78
65 til 69	H	40.87	8.17	16.35	24.52	32.70	32.70	H	23.60	4.72	9.44	14.16	18.88	18.88
	V	36.57	7.31	14.63	21.94	29.26	29.26	V	23.38	4.68	9.35	14.03	18.70	18.70
70 til 74	H	37.48	7.50	14.99	22.49	29.98	29.98	H	25.84	5.17	10.34	15.50	20.67	20.67
	V	35.49	7.10	14.20	21.29	28.39	28.39	V	22.92	4.58	9.17	13.75	18.34	18.34
75+	H	32.76	6.55	13.10	19.66	26.21	26.21	H	19.40	3.88	7.76	11.64	15.52	15.52
	V	28.59	5.72	11.44	17.15	22.87	22.87	V	17.64	3.53	7.06	10.58	14.11	14.11

Gilbertson, L. and Barber-Lomax, S. (1994), 'Power and Pinch Grip Strength Recorded Using the Hand-Held Jamar Dynamometer and B+L Hydraulic Pinch Guage: British Normative Data for Adults', *British Journal of Occupational Therapy*, 57 (12), pp. 483-488.

C. Schanke 01/11/2022 © Copyright of Chelsea and Westminster NHS Foundation Trust (01/03/2010)
